# Are computer and cell phone use associated with body mass index and overweight? A population study among twin adolescents

**DOI:** 10.1186/1471-2458-7-24

**Published:** 2007-02-26

**Authors:** Hanna-Reetta Lajunen, Anna Keski-Rahkonen, Lea Pulkkinen, Richard J Rose, Aila Rissanen, Jaakko Kaprio

**Affiliations:** 1 Dept of Public Health, University of Helsinki, P.O. Box 41, 00014, Helsinki, Finland; 2 Obesity Research Unit, Dept of Psychiatry, Helsinki University Central Hospital, Helsinki, Finland; 3 Dept of Epidemiology, Mailman School of Public Health, Columbia University, New York, USA; 4 Dept of Psychology, University of Jyväskylä, Jyväskylä, Finland; 5 Dept of Psychological & Brain Sciences, Indiana University, Bloomington, IN, USA; 6 Dept of Mental Health and Alcohol Research, National Public Health Institute, Helsinki, Finland

## Abstract

**Background:**

Overweight in children and adolescents has reached dimensions of a global epidemic during recent years. Simultaneously, information and communication technology use has rapidly increased.

**Methods:**

A population-based sample of Finnish twins born in 1983–1987 (N = 4098) was assessed by self-report questionnaires at 17 y during 2000–2005. The association of overweight (defined by Cole's BMI-for-age cut-offs) with computer and cell phone use and ownership was analyzed by logistic regression and their association with BMI by linear regression models. The effect of twinship was taken into account by correcting for clustered sampling of families. All models were adjusted for gender, physical exercise, and parents' education and occupational class.

**Results:**

The proportion of adolescents who did not have a computer at home decreased from 18% to 8% from 2000 to 2005. Compared to them, having a home computer (without an Internet connection) was associated with a higher risk of overweight (odds ratio 2.3, 95% CI 1.4 to 3.8) and BMI (beta coefficient 0.57, 95% CI 0.15 to 0.98). However, having a computer with an Internet connection was not associated with weight status. Belonging to the highest quintile (OR 1.8 95% CI 1.2 to 2.8) and second-highest quintile (OR 1.6 95% CI 1.1 to 2.4) of weekly computer use was positively associated with overweight. The proportion of adolescents without a personal cell phone decreased from 12% to 1% across 2000 to 2005. There was a positive linear trend of increasing monthly phone bill with BMI (beta 0.18, 95% CI 0.06 to 0.30), but the association of a cell phone bill with overweight was very weak.

**Conclusion:**

Time spent using a home computer was associated with an increased risk of overweight. Cell phone use correlated weakly with BMI. Increasing use of information and communication technology may be related to the obesity epidemic among adolescents.

## Background

Prevalence of overweight is rapidly increasing among children and adolescents. As defined by the International Obesity Task Force criteria, the prevalence of obesity is estimated to be about 10% throughout the world among 5–17-year-olds and dramatically higher in developed countries, reaching about 30% in the Americas [1]. Genetic predisposition to obesity is high, with the heritability of BMI estimated to be 50–90% [2]. However, also environmental factors are important: while genes may predispose a child to weight gain, environmental factors can foster development of obesity.

Increasingly sedentary leisure activities are considered one of the main reasons for the obesity epidemic [1]. According to a meta-analysis based on 52 independent samples [3], there is a statistically significant relationship between TV viewing and body fatness among 3–18-year-olds. Conversely, there is an inverse association between sports/physical activity and weight status in cross-sectional studies [4-8].

Although sedentary leisure time activities involving information technology use have spread rapidly among children and adolescents, the association of weight status with computer and cell phone use has not been thoroughly investigated. Computer use was positively associated with BMI [9] and overweight [10] among adolescent girls but not among boys. Video/computer games were not significantly associated with overweight among children and teenagers [3,10], nor was video game/computer time among children [11]. One study found a positive association between electronic games and weight status among boys and girls [12] and another among girls only [13]. We were not able to find any studies on the associations between cell phone use and weight status. Cell phone use is, however, positively associated with some health-compromising behaviors, such as tobacco, snuff, and alcohol use [14], which in turn are related to BMI and sedentary lifestyle [15].

Given the paucity of data on the effects of information and communication technology on adolescent health, we studied the relation of the use of these technologies with weight status. Over 90% of Finnish 15–19-year-olds owned a personal cell phone at the end of 2002 [16]. Cell phone use started to spread to all age groups during the mid 90s [17]. The rapid increase of cell phone ownership has multiplied the opportunities to spend time on the phone and total phone time has steadily increased in Finland [19]; this may reduce time spent in physically active leisure activities. Therefore, we hypothesized that cell phone use would be associated with BMI and overweight.

At 1999–2000, boys aged 15–18 y spent in average 50 and girls 10 minutes using a computer during school days. During non-school days, boys spent almost 80 minutes and girls about 20 minutes using computers [18]. We hypothesized that overweight and BMI would be influenced by both computer use hours and home computer ownership, with the highest computer use hours being among adolescents having a home computer with an Internet connection. We tested these hypotheses cross-sectionally in a population sample of 17-year-old girls and boys.

## Methods

### Participants

This study is a part of an ongoing Finnish twin-family study (*FinnTwin12*) of the behavioral development and health habits of the five birth cohorts 1983–1987 [20,21]. Local ethics committees and internal review boards in Helsinki and Indiana reviewed and approved the study protocol. We obtained a written informed consent from participating twin families.

The data in the present analyses were derived from self-report questionnaires returned by the parents (N = 2736 mothers and 2636 fathers, participation rates 94% and 88%) and their twin children, who responded at ages 11–12, 14, and 17.5 y. Computer and cell phone use were assessed at age 17.5 y, so only data on the twins from that wave (N = 4098, participation rate 90%) and that from their parents at baseline (when the twins were 11–12 y) were used for the analyses here reported. This wave of questionnaires was carried out from the autumn of 2000 to late spring of 2005.

### Measures

#### Overweight

Using self-reported height and weight, we calculated the body mass indices (weight/height^2^) of adolescents. To eliminate outliers resulting from data errors, we used least median squares regression fit method [22,23] and the *zanthro *function in Stata (Version 8.0) to transform BMI data to z-scores based on exact age at response; participants with z-scores over 5 or under -5 were not included in the analyses. The reference data used were the 1990 British Growth Reference [24]. After exclusion of outliers and participants with missing height or weight (70 adolescents, 1.7%), the final sample consisted of 4098 adolescents. Adolescents who were excluded from the analyses did not differ in terms of computer ownership or computer use, but their cell phone bills were smaller when compared to participants who were included in the analyses. Adolescents with data missing on computer ownership or computer or cell phone use did not differ in BMI compared to those with data on these items. There was no difference between the mean BMI of 14-year-olds participating in the 17 y questionnaire and the mean BMI of the non-participants.

Overweight was defined according to Cole et al. [25] using age- and sex-specific cut-off points that correspond to adult cut-off points for overweight (25 kg/m^2^) and obesity (30 kg/m^2^). The function *zbmicat *in Stata was used to categorize adolescents as obese, overweight or normal weight. Children defined as obese according to Cole at al. were analyzed in the overweight children's group, because there were too few obese children for separate analyses of obesity (Table 1). Participants who were 18 y or older (4.6%) when responding to the questionnaire, were categorized using adult cut-off points for overweight and obesity.

**Table 1:** Sample Characteristics

	Boys	Girls	Boys and girls
N	1981	2117	4098
mean age, SD	17.6 (0.24)	17.6 (0.27)	17.6 (0.26)
mean BMI, SD	21.8 (3.0)	21.0 (3.0)	21.4 (3.0)
prevalence of overweight and obesity (%)	11.9	7.9	9.8
prevalence of overweight (%)	9.4	6.6	8.0
prevalence of obesity (%)	2.4	1.3	1.9

#### Computer and cell phone ownership and use

The independent study variables were both categorical (cell phone bill and computer ownership) and continuous (computer use hours). Participants were asked whether or not they had a computer at home. Because we thought that having an Internet connection could increase computer use hours, we also asked whether their home computer had an Internet connection. Weekly computer use hours for school/work and recreation were then assessed, and five quintile-based categories of computer use were formed. Distribution of computer use hours was extremely skewed; therefore the variable was categorized to ensure sufficient sample sizes. Cell phone use was addressed via the participants' monthly cell phone bill, with the following response options: no cell phone, monthly total fewer than 10 euros, 10 – 20 euros, 20 – 35 euros, 35 – 85 euros, and over 85 euros (a euro being about 1.3 USD in August 2006). The two highest categories were combined, because there were too few (twenty) observations in the last category.

### Potential confounders

Participants were asked how often they engage in physical exercise or sports in their leisure time; response options were: 1) not at all; 2) more seldom than once in a month; 3) 1–2 times in a month; 4) about once in a week; 5) 2–3 times in a week; 6) 4–5 times in a week; 7) almost every day. Mean days of physical exercise in a year were calculated based on the responses and divided then by the number of weeks in a year to get a semi-continuous variable of mean exercise days in a week.

Parental education was divided into parents who had completed the academic high school ("high education") versus those who had completed the mandatory school requirement (9 years) only or had had vocational education ("low education"). Education levels of both mothers and fathers were included in the analysis. A missing answer was defined as a third category of the education variables. Parents' occupation was also included. Seven occupational categories were formed: 1) self-employed persons; 2) upper-level employees; 3) lower-level employees; 4) manual workers; 5) students; 6) pensioners, and 7) others (unemployed, not classified elsewhere) [26,27]. The last three categories were collapsed into one in the analyses because there were too few observations. The first category was used as a reference group in the analyses.

### Data analysis

Linear regression models were used to study the associations between computer and cell phone use and BMI and logistic regression models to study the associations with overweight. The overall significance of variables was tested using a likelihood ratio test based on regression models with and without the variable in question. Because BMI distribution was only slightly skewed to the right and also because of the much more straightforward interpretation of the results, we decided to present results of linear regression using raw BMI. However, use of log-transformed values would have had only a minor effect on the results, with no changes in inference. To obtain correct confidence intervals, the effect of potential non-independence of observations (i.e. twins within twin pairs) was taken into account by computing robust estimators of variance and correcting for clustered sampling of families [28]. The models were adjusted for gender, physical exercise (times in a week), and mother's and father's education levels and occupational class. The association of a cell phone bill with weight status was analyzed in two ways: 1) the variable was used as a 'continuous' variable (categories numbered consecutively 1–5 in a trend test) and 2) the monthly cell phone bill was used as a categorical variable, a reference group being the most prevalent category (20–35 euros). The reference category was chosen from the middle of the scale. As the category with the largest n of subjects it was the most normative group, which also provided statistical stability.

## Results

The prevalence of overweight among adolescents was 8.0% and the prevalence of obesity 1.9% (Table 1). Prevalences of overweight and obesity were about 50% higher in boys than in girls (OR compared to girls 1.56, 95% CI 1.24 to 1.96). Of all the adolescents, about 86% had a computer at home and 94% owned a cell phone (Table 2).

**Table 2:** Information and communication technology use *

		N (%), boys	N, (%) girls	N, (%) boys and girls
Computer at home	No	226 (11)	323 (15)	549 (13)
	Yes			
	without Internet	346 (17)	316 (15)	662 (16)
	with Internet	1409 (71)	1478 (70)	2887 (70)
Cell phone bill in a month (euros)	No cell phone	140 (7)	114 (5)	254 (6)
	< 10	295 (15)	173 (8)	468 (11)
	10 – 20	711 (36)	635 (30)	1346 (33)
	20 – 35	647 (33)	910 (43)	1557 (38)
	>35	188 (9)	284 (13)	472 (12)
Weekly computer use for school, work, and recreation (hours)	0–1	246 (13)	586 (28)	832 (20)
	>1–3	307 (16)	625 (30)	932 (23)
	>3–6	388 (20)	465 (22)	853 (21)
	>6–12	419 (22)	287 (14)	706 (17)
	>12–115	580 (30)	135 (6)	715 (18)

* Percentages do not all add up to 100% in this table because of rounding. The exact numbers are used in the analyses.

The proportion of adolescents without a cell phone decreased across time during data collection, from 12% at 2000–2001 to 1% at 2004–2005. The proportion of adolescents without a computer decreased from 18% to 8%, while the proportion of adolescents with a computer and an Internet connection increased from 61% to 81%. (Table 3). Weekly computer use time was 2.8 hours (95% CI 2.4 to 3.2) among adolescents not having a home computer, 6.0 h (95% CI 5.3 to 6.7) among those having a computer without an Internet connection, and 8.9 h (95% CI 8.5 to 9.4) among those having a computer and an Internet connection.

**Table 3:** Computer and cell phone ownership by year

	Computer at home, n (%)			Cell phone ownership
Year (cohort)	No	Without Internet	With Internet	No cell phone, n (%)

2000–2001 (1983)	181 (18)	214 (21)	611 (61)	120 (12)
2001–2002 (1984)	132 (16)	144 (17)	557 (67)	58 (7)
2002–2003 (1985)	100 (12)	131 (16)	589 (72)	41 (5)
2003–2004 (1986)	79 (11)	102 (14)	571 (76)	25 (3)
2004–2005 (1987)	57 (8)	71 (10)	559 (81)	10 (1)

### Linear regression models

The results of linear regression models are detailed in Table 4. The results from the analysis where home computer ownership, computer use hours, and a continuous cell phone bill variable were analyzed in one linear model in a combined analysis and the results from likelihood ratio tests are reported below. There was a difference in BMI depending on computer ownership (likelihood ratio test p = 0.011). Adolescents owning a computer without an Internet connection had a greater BMI than those without a computer (0.57 kg/m^2 ^higher, 95% CI 0.15 to 0.98; and 0.47, 95% CI 0.05 to 0.90 in a combined analysis). BMI of adolescents having a computer with an Internet connection did not differ from those not having a computer at all (Table 4). Computer use hours were not associated with BMI (likelihood ratio test p = 0.42). There was a positive linear trend between the amount of a cell phone bill and BMI when a cell phone bill was used as a 'continuous' variable in a trend test (beta 0.18, 95% CI 0.06 to 0.30; the result remained the same in a combined analysis). When cell phone bill was used as a categorical variable, there was a difference in BMI depending on the cell phone bill (likelihood ratio test p = 0.004). As the regression coefficients increased in an ordered manner (Table 4), the change was best represented by the 'continuous' cell phone bill measure (the result reported above). All models were adjusted for the same confounding factors, of which female gender (beta -0.81, 95% CI -1.0 to -0.58) and father's high education (beta -0.37, 95% CI -0.73 to -0.01), were associated with a decreased risk of overweight, while when compared to self-employed women, other mothers' occupational categories did not differ in BMI with the exception of lower-level employees (beta -0.51, 95% CI -1.00 to -0.03).

**Table 4:** Associations of computer and cell phone use with BMI at 17 y

		Mean BMI (SD)	Linear regression, adjusted for gender, physical exercise, and parents' education	
			
			Beta coefficient, 95% confidence interval	p-value
Computer at home	No	21.3 (2.9)	-	-
	Yes			
	without Internet	21.6 (3.0)	0.57 (0.15 to 0.98)	0.007
	with Internet	21.3 (3.1)	0.23 (-0.09 to 0.56)	0.16
Weekly computer use for school, work, and recreation (hours)	0–1	21.1 (3.0)	-	-
	>1–3	21.2 (2.9)	0.10 (-0.21 to 0.41)	0.52
	>3–6	21.3 (2.9)	0.12 (-0.20 to 0.44)	0.46
	>6–12	21.6 (3.0)	0.31 (-0.0.38 to 0.65)	0.081
	>12–115	21.7 (3.4)	0.25 (-0.14 to 0.65)	0.21
Cell phone bill	(continuous)	-	0.18 (0.06 to 0.30)	0.003
Cell phone bill in a month (euros)	No cell phone	21.0 (3.4)	-0.53 (-1.09 to 0.04)	0.068
	< 10	21.3 (3.6)	-0.20 (-0.60 to 0.21)	0.34
	10 – 20	21.3 (2.9)	-0.25 (-0.49 to -0.006)	0.045
	20 – 35	21.4 (2.9)	-	-
	35 – 85	21.7 (2.9)	0.29 (-0.04 to 0.63)	0.085

### Logistic regression models

The results of logistic regression models are shown in Table 5. The results from the analysis where home computer ownership, computer use hours, and a categorical cell phone bill variable were analyzed in one logistic model in a combined analysis and the results from likelihood ratio tests are reported below. There was a difference in the proportion overweight depending on computer ownership (likelihood ratio test p < 0.001). The proportion overweight among adolescents owning a computer without an Internet connection was greater than the proportion among those without a computer (OR 2.3, 95% CI 1.4 to 3.8 (Table 5); and in a combined analysis: OR 1.9, 95% CI 1.1 to 3.3). However, the proportion overweight among adolescents having a computer and an Internet connection did not differ from those not having a computer (Table 5). There was an association between time spent on computer use and the probability of being overweight (likelihood ratio test p = 0.002). Higher proportions of those who spent longer times on the computer were overweight, with those using the computer for more than 12 hours a week being 1.8 times and those using it 6 to 12 hours 1.6 times as likely to be overweight as those using the computer less than 1 hour per week (Table 5). In a combined analysis the equivalent results were OR 1.9 (95% CI 1.2 to 2.9) for more than 12 hours and OR 1.6 (95% CI 1.1 to 2.5) for 6 to 12 hours. Cell phone bill did not have a linear trend in association with the risk of overweight. The ordinal categories showed some effect (likelihood ratio test p = 0.028) but there was no clear pattern (Table 5). The results for cell phone bill were similar in a combined analysis. All models were adjusted for the same confounding factors, of which female gender (OR 0.75, 95% CI 0.56 to 0.99), father's high education (OR 0.61, 95% CI 0.39 to 0.96), and increased frequency of physical exercise per week (OR 0.88, 95% CI 0.83 to 0.93) were associated with a decreased risk of overweight, while major occupational classes were not.

**Table 5:** Associations of computer and cell phone use with overweight at 17 y

		Overweight, n (%)	Logistic regression, adjusted for gender, physical exercise, and parents' education and occupational category
Computer at home	No	50 (9)	1.0
	Yes		
	without Internet	84 (13)	2.26 (1.35 to 3.76)
	with Internet	269 (9)	1.51 (0.94 to 2.42)
Weekly computer use for school, work, and recreation (hours)	0–1	23 (10)	1.0
	>1–3	32 (11)	0.95 (0.62 to 1.46)
	>3–6	36 (9)	1.19 (0.79 to 1.81)
	>6–12	51 (12)	1.61 (1.07 to 2.41)
	>12–115	88 (15)	1.81 (1.20 to 2.75)
Cell phone bill	(continuous)	-	1.02 (0.90 to 1.16)
Cell phone bill in a month (euros)	No cell phone	31 (12)	1.25 (0.76 to 2.06)
	< 10	50 (11)	1.05 (0.71 to 1.56)
	10 – 20	106 (8)	0.73 (0.54 to 0.98)
	20 – 35	159 (10)	1.0
	>35	57 (12)	1.23 (0.85 to 1.78)

## Discussion

During 2000–2005, the proportion of Finnish 17-year-old adolescents without a home computer steadily decreased from 18% to 8%. Weekly hours of computer use were positively associated with an increased risk of overweight. The proportion of adolescents without a cell phone decreased from 12% to just 1% during our study period. We found only a weak positive association between BMI and the monthly total cell phone bill.

How could computer and cell phone use affect weight status? An inverse association of physical activity with BMI and obesity among adolescents has been confirmed in many studies [4-8]. Television viewing time, another important behavioral correlate of obesity [3], has been hypothesized to affect weight via three main pathways: by 1) taking time away from more physically active behaviors; 2) increasing energy intake by snacking while viewing and by television commercials; and 3) decreasing the metabolic rate [29]. Conversely, video/computer game playing has been associated with increases in various metabolic and physiologic variables [30,31]. Similarly to TV viewing, computer and cell phone use may increase the proportion of time devoted to sedentary activities. The rapid increase of cell phone ownership has multiplied the opportunities to spend time on the phone and total phone time (cell phone & landline combined) has steadily increased in Finland [19]. When a cell phone call, text message, or e-mail replaces a trip to a friend's house or eliminates the need to run an errand, daily physical activity is reduced.

Computer use has been found to be positively associated with weight status in adolescent girls, but not in boys among 4746 American teenagers in 1998–99 [9] and among 6515 Finnish teenagers in 2001 [10]. Since then, home computers have become almost ubiquitous: we thus wished to reassess their association with overweight and BMI. In this study, we observed that computer use was associated with weight status independent of gender, with significant positive associations between computer use and overweight in boys and between computer use and BMI among girls (gender-specific results not shown).

There were some incongruences in the results considering computer ownership; adolescents having a home computer with an Internet connection had the highest computer use hours, but their BMI and risk of overweight did not significantly differ from those not having a home computer. However, adolescents who had a home computer not connected to the Internet had a significantly higher BMI and a higher risk of overweight compared to adolescents not having a home computer at all. There are many possible explanations for this: either computer use might be somehow different in the presence of an Internet connection, or people with and without an Internet connection might recall their computer use hours differently, or the differences could be due to residual confounding. In any case, these questions merit further investigation.

The results were partly different when using BMI vs. overweight as an outcome, which implies that the distribution of BMI is probably different in different categories of the predictors. BMI had no overall association with physical exercise, while overweight was strongly negatively associated with exercise. Among normal-weight and underweight adolescents, differences in fat-free muscle mass can explain a large part of the BMI differences, though a high BMI appears a good indicator of excess fat mass [32].

The main limitation of this study is its cross-sectional design, which does not allow confident causal inferences. We cannot exclude the possibility that overweight or an increase in BMI increase computer or cell phone use. Prospective studies are needed to confirm our findings. It is also important to consider other potential confounders that may influence our results, such as eating habits, the availability of pocket money, and certain personality traits (impulse control, sociability). Future studies should address the association of new technology use and weight status by carefully adjusting for nutritional intake, multiple socioeconomic factors, and other potential confounders, preferably in a prospective study design.

Our study is also limited by its reliance on self-reported behaviors. Objective tracking of patterns of computer and cell phone use would yield more reliable results, but was not feasible in this large cohort of adolescents spread over a large and sparsely populated geographical area. Also, verifying billing information or actual time spent on a cell phone from cellular network providers would enhance reliable results, but was impossible because of privacy protection requirements. In Finland, the monthly cell phone bill is a relatively good proxy measure for actual time spent on the phone, because outgoing calls are typically priced on a per minute basis and text messages on a per item basis; incoming calls and text messages are usually free of charge. During the study period charging for cell phone use was uniform in Finland although the prevailing practice is about to change.

Self-reported heights and weights are also potential sources of bias. Reliance on self-report underestimates the true prevalence of overweight [33,34]. Among 418 boys and girls whose mean age was 16.30 y, it led to under-estimation of overweight by 4.8% and obesity by 1.6% [33]. However, our main goal was not to estimate prevalence of overweight but to study the associations between information and technology use and BMI/overweight. Only if self-report bias of weight and height differs by computer and cell phone use, would the associations observed in our study be biased.

Finally, the generalizability of results from a study of twins to non-twin individuals may be questioned. But differences specific to twins in anthropometric, social, and physical   activity measures during late adolescence are relatively minor [15][35-37], and they are unlikely to affect the relationship of computer and cell phone use with the risk of overweight. It is possible that hours of computer use are reduced in twin families because many families share just one home computer. However, this would closely resemble the situation of families with many non-twin children close in age.

The strengths of our study include large and population-based study sample, its high participation rates, and the availability of information from the parents. The data are very recent, reflecting developments in the 21^st ^century, particularly the widespread adaptation of information and communication technology. Finland is well suited for this kind of study given its early adoption and high penetration of communication technology use.

## Conclusion

This study confirms that the global epidemic of overweight and the use of new technologies may be related phenomena: weight status is associated with computer and possibly cell phone use. However, given that computer and cell phone use constitutes only a small part of adolescents' leisure time, broader patterns of leisure time use should be addressed in a longitudinal setting to obtain a comprehensive view of their relationship with weight status. Future research should confirm and expand our findings to ensure that the use of modern communication technology does not have undesired health effects among children and adolescents.

## Abbreviations

Body mass index (BMI), odds ratio (OR), beta coefficient (beta)

## Competing interests

The author(s) declare that they have no competing interests.

## Authors' contributions

H-RL carried out the statistical analyses and drafted the manuscript. AK-R participated in statistical analyses and interpretation of data and helped to draft the manuscript. LP and RJR made substantial contributions to conception and design of data and were involved in drafting and revising the manuscript. AR made substantial contributions to interpretation of data and was involved in drafting and revising the manuscript. JK made substantial contribution to conception, design, and acquisition of data, participated in statistical analyses, interpretation of data, and was involved in drafting and revising the manuscript. All authors read and approved the final manuscript.

## Pre-publication history

The pre-publication history for this paper can be accessed here:


